# A New Method for Automatic Detection of Defects in Selective Laser Melting Based on Machine Vision

**DOI:** 10.3390/ma14154175

**Published:** 2021-07-27

**Authors:** Zhenqiang Lin, Yiwen Lai, Taotao Pan, Wang Zhang, Jun Zheng, Xiaohong Ge, Yuangang Liu

**Affiliations:** 1 College of Materials Science and Engineering, Xiamen University of Technology, Xiamen 361024, China; zhenqianglin@3dmetalwerks.com (Z.L.); taotaopan@3dmetalwerks.com (T.P.); wangzhang@3dmetalwerks.com (W.Z.); 2 College of Electrical Engineering and Automation, Xiamen University of Technology, Xiamen 361024, China; yiwenlai@3dmetalwerks.com; 3 College of Chemical Engineering, Huaqiao University, Xiamen 362021, China

**Keywords:** selective laser melting, powder spreading defect, machine vision, classifier

## Abstract

Selective laser melting (SLM) is a forming technology in the field of metal additive manufacturing. In order to improve the quality of formed parts, it is necessary to monitor the selective laser melting forming process. At present, most of the research on the monitoring of the selective laser melting forming process focuses on the monitoring of the melting pool, but the quality of forming parts cannot be controlled in real-time. As an indispensable link in the SLM forming process, the quality of powder spreading directly affects the quality of the formed parts. Therefore, this paper proposes a detection method for SLM powder spreading defects, mainly using industrial cameras to collect SLM powder spreading surfaces, designing corresponding image processing algorithms to extract three common powder spreading defects, and establishing appropriate classifiers to distinguish different types of powder spreading defects. It is determined that the multilayer perceptron (MLP) is the most accurate classifier. This detection method has high recognition rate and fast detection speed, which cannot only meet the SLM forming efficiency, but also improve the quality of the formed parts through feedback control.

## 1. Introduction

As one of the most promising technologies in metal additive manufacturing, selective laser melting (SLM) mainly uses the idea of layer-by-layer accumulation to construct metal parts [1]. SLM technology can produce complex parts that cannot be formed by traditional manufacturing technology, so it has a broad application prospect in aerospace, medical, molds, and other fields [2,3,4]. 

It is undoubtedly the ultimate goal of SLM technology to directly produce metal parts with excellent performance and quality, but the SLM forming process is a complex physical and chemical process. The quality of SLM forming parts is influenced by many factors, including laser parameters (laser power, spot size, and laser wavelength), scanning parameters (scanning strategy, scan spacing, and scanning speed), environmental conditions (oxygen content and humidity), and material conditions (thick, spread powder, and metal powder particle size) [5,6,7,8]. 

Therefore, it is very necessary to monitor the formation process of SLM to ensure the quality of finished products. According to the selected laser melting process steps, the selected laser melting process detection can be divided into the powder spreading process detection, powder bed detection, melting process detection, and molten layer detection. 

For example, Reinarz et al. [9] detected the stability of the powder spreading device by the principle that the powder spreading device would produce a speed change when passing through the raised molten layer. Tom Craeghs et al. [10] used the column-wise cross-sectional view of the average value of the gray pixels in each row of the powder bed image to detect whether the scraper was damaged. Clijsters et al. [11] used information such as molten pool heat, molten pool area, molten pool length, molten pool width, etc. with good performance of formed parts as reasonable data, compared and evaluated the collected molten pool information with a reasonable data interval, and then identified the melting abnormal pool and assess the forming quality of parts. Foster et al. [12] collected molten layer images through industrial cameras, used edge detection algorithms to extract the contours of the molten layer, analyzed and processed the original images of each layer, superimposed them into a three-dimensional model, and detected defects by evaluating three-dimensional data. 

To monitor the SLM forming process effectively, machine vision inspection is a safe, reliable, and highly automated non-contact inspection technology that can mimic human eye functions through industrial cameras and extract effective information from various environments [13]. The application of this technology in the industrial field is relatively large, mainly involving the detection of surface defects in metals, fabrics, glass, electronic devices, etc. [14,15,16,17].

Among the SLM process steps, the quality of the powder spreading has a huge impact on the final quality of SLM molded parts. Packing quality refers to the smoothness, uniformity, and whether there are impurities in the surface of the powder during each laying operation by SLM equipment. The most concern in the SLM powder coating process is the flatness and layer thickness error of the powder coating surface. Poor flatness can easily cause incomplete melting or over-melting when the laser is applied to the convex or concave surface of the powder coating surface. If the thickness is too thick, the layers may not be fully fused together. If the thickness is too thin, the thermal stress may be too large. Therefore, when the surface of the powder coating is defective, the surface quality of the single-layer processing will deteriorate. The accumulation of the single-layer surface with poor quality will cause pores, cracks, and surface spheroidization in the final formed part, which will seriously affect the performance of the part [18,19,20]. In order to further improve the performance and quality of SLM forming parts, it is necessary to ensure the quality of each layer of the powder coating surface during the SLM forming process. 

In this paper, to solve the problem of poor performance of forming part caused by powder spreading defect, the machine vision inspection technology is used to monitor the powder spreading condition in the material condition factors, the construction of SLM powder spreading defect collection system, and the detection algorithm of SLM powder spreading defects are mainly studied. 

## 2. Experiment Setup and Powder Spreading Defect

### 2.1. Experiment Setup

**Figure 1:**
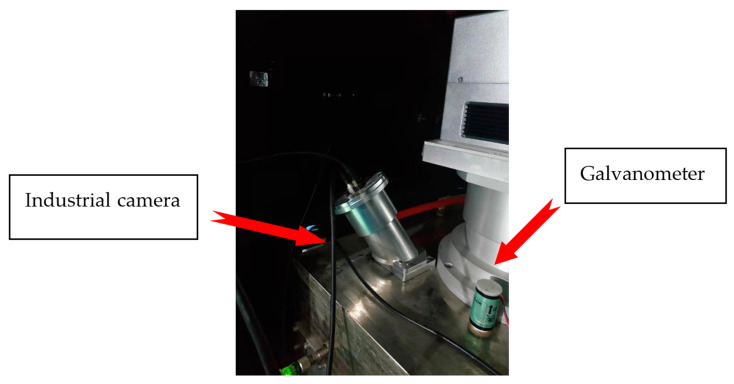
Industrial camera installation location.

As the laser galvanometer system (Scancube 3, Scanlab, Siemensstr. 2a82178 Puchheim Germany) is already installed directly above the forming bin of the selective laser melting equipment (WXL-120, 3D Metalwerks Co. Ltd., Xiamen, China), the image of the powdered surface can only be collected by the side axis. The specific installation location is shown in Figure 1. In order to improve the resolution capability of the system, because the size of the forming bin of the SLM equipment used in this system is 120 × 120 × 100 (mm), and to enable the detection system to resolve at least 0.5 mm of details, each detail needs to be composed of 4–8 pixels. This system takes six pixels corresponding to a 0.5 mm defect, so the number of pixels in the length and width direction of the forming chamber needs to be greater than 1440, and due to the angle of the camerac, the powder bed of the forming cylinder will appear on the image in a diamond shape, so the final pixels in all directions of the camera number needs to be greater than that $1440\times \sqrt{2}\approx 2037$, so we used a Hikvision MV-CA050-10GM camera with sensor type IMX264, pixel size 3.45 μm × 3.45 μm, sensor area 2/3”, resolution 2448 × 2448, frame rate 23.5 fps, and camera interface gigabit ethernet. 

After the industrial camera is selected, high-quality images cannot be acquired without a matching lens. The function of the camera lens is similar to that of the crystalline lens. It mainly focuses the light reflected by the object on the sensor. The distance from the camera to the powder spreading surface of this system is 215 mm, thus the magnification is β = 6.6/$120\sqrt{2}$ = 0.0389, and the focal length of the objective lens is 215/(1 + 1/β) = 8.05 mm. Therefore, we chose the FL-CC0814A-2M Ricoh 8 mm lens with format size 2/3”, focal length 8 mm, iris range 1.4–16, and minimum object distance 0.1 mm. 

**Figure 2:**
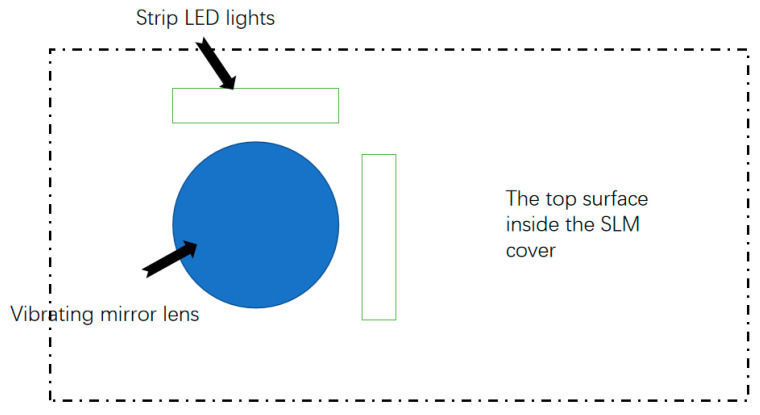
Light-Emitting Diode (LED) light installation position.

In this work, the strip Light-Emitting Diode (LED) light is used as the light source of the detection system. The installation position of the strip LED light source is shown in Figure 2. 

### 2.2. SLM Powder Spreading Defect Type

In the SLM powder spreading process, defects are easily formed on the powder coating surface due to improper selection of process parameters or wear of the scraper strips. Several common powder spreading defects mainly include the following.

**Figure 3:**
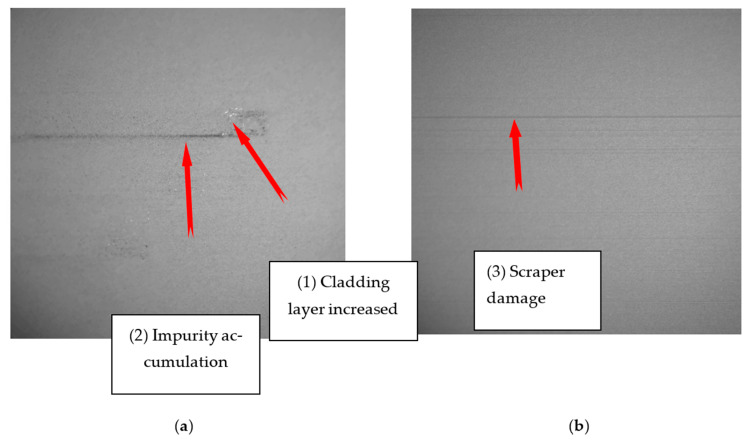
SLM powder spreading defect: (**a**) Defects of cladding (1) and impurity accumulation (2). (**b**) Scraper damage defects (3).

(1) Cladding layer increased. Due to the phenomenon of spheroidization, warpage, and incomplete powder melting during the molding process of the selected laser melting equipment, it is difficult to control the molding process. The spheroidization phenomenon occurs when the surface of the metal solution turns into a spherical surface under the action of interfacial tension between the molten metal and the surrounding powder particles. Therefore, spheroidization often causes unevenness on the surface of the part, which leads to the phenomenon of local high. In the process of powder spreading, it is difficult for the metal powder to cover the local high area, as shown in Figure 3 (1). 

(2) Impurity accumulation. Due to the excessive energy of the molten pool, the liquid metal evaporates to produce black smoke impurities. During the laser scanning process, due to the influence of the laser scanning direction and the laser scanning speed, part of the black smoke impurities falling on the metal surface cannot be brought into the airflow generated by the fan into the gas circulation system, and thus black smoke impurities accumulate. When the powder spreading device is activated, the scraper will drive the impurities and form this kind of defect, as shown in Figure 3 (2). 

(3) Scraper damage. Due to the friction of the scraper with the higher part of the cladding layer for a long time, the surface of the scraper is partially missing. When the powder spreading device is activated, part of the metal powder driven by the scraper will leak out from the position where the scraper strip is missing, forming a scraper damage defect, as shown in Figure 3 (3). 

As impurity accumulation and scraper damage appear as stripes on the image, these two defects are collectively referred to as strip defects (3). 

### 2.3. Defect Overlap

For the overlap of defects, there is only the probability that the scratch damage and impurities will overlap in the above defects, and the impurity defects will be disposed of in the first powder laying, while the scratch defects will not. The cladding layer is raised, because the detection threshold is different, it is not necessary to classify the calculation together, so defect overlap has no effect on the overall detection. 

## 3. Image Processing Method

### 3.1. Perspective Transformation

In this paper, the industrial camera mainly adopts the side-axis method, so the collected powder spreading images have some linear distortion. Linear distortion can easily cause distortion of the powder spreading image and increase the difficulty of the defect detection, so corresponding measures should be taken to correct it. 

The process of correction is mainly to project each pixel of the image to a new plane at one time. The specific equation is as follows:

(1) $$\left[\begin{array}{c} u_{corr}\\ v_{corr}\\ z_{corr} \end{array}\right]=\left[\begin{array}{l} h_{11}\;h_{12}\;h_{13}\\ h_{21}\;h_{22}\;h_{23}\\ h_{31}\;h_{32}\;h_{33} \end{array}\right]\cdot \left[\begin{array}{c} u\\ v\\ 1 \end{array}\right]$$

In the equation, (u,v) is the original coordinates of P, $\left(u_{\mathrm{corr}},v_{corr},z_{corr}\right)$ is the coordinates of $P_{corr}$, ($h_{11}$, $h_{12}$; $h_{21}$, $h_{22}$) is the linear transformation matrix, ($h_{13}$; $h_{23}$) is the translation matrix, ($h_{31}$, $h_{32}$) is the perspective matrix, because the final corrected image is located in the two-dimensional plane, so the coordinates of $P_{corr}$ are divided by $z_{corr}$, and the transformation equation is as follows:

(2) $$\left\{ \begin{array}{l} {u^{\prime }}_{corr}=\frac{h_{11}u+h_{12}v+h_{13}}{h_{31}u+h_{32}v+1}\\ {v^{\prime }}_{corr}=\frac{h_{21}u+h_{22}v+h_{23}}{h_{31}u+h_{32}v+1}\\ {z^{\prime }}_{corr}=1 \end{array}\right.$$

In the equation, $\left({u^{\prime }}_{corr},{v^{\prime }}_{corr},1\right)$ is the final corrected coordinate. 

**Figure 4:**
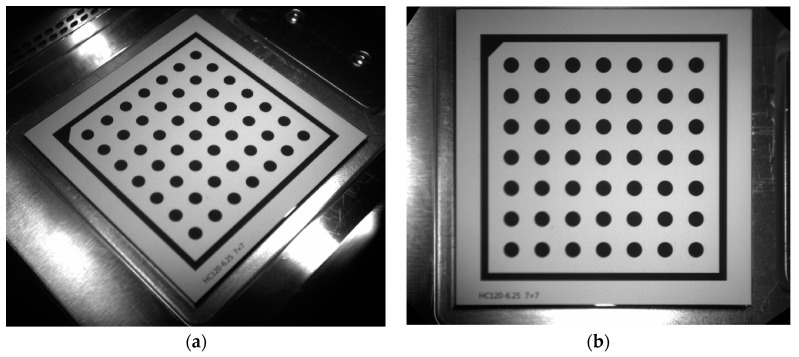
The perspective transformation effect: (**a**) Perspective transformation before. (**b**) After the perspective transformation.

It is not difficult to see from Equation (2) that only four points are needed to list eight equations and find eight unknown quantities of the correction transformation matrix. The perspective transformation effect is shown in Figure 4. 

### 3.2. The Steps of Defect Extraction

This article mainly describes the increased defects in the cladding layer and the stripe defects, the specific steps can be divided into three steps.

Step 1: Eliminate the effects of light. 

It can be seen from Figure 3 that the image of the powdered surface is affected by the LED light, resulting in uneven illumination of the image, so measures should be taken to eliminate the influence of illumination and avoid increasing the difficulty of image processing in the later stage. Specific steps to eliminate the influence of light:

(1) Calculate the average gray value of the entire image.
(2) Perform average filter on the image, mainly using a 200 × 200 filter template.
(3) Subtract the original image from the average filtered image and add the gray average value of the entire image.

The above steps can be expressed as

(3) $$G_{\mathrm{out}}=G_{\mathrm{in}}-G_{mean}+v$$

In the equation, $G_{\mathrm{out}}$ is the output image, $G_{\mathrm{in}}$ is the input image, $G_{mean}$ is the average filtered image, and v is the average of the entire image. 

For example, enter $G_{\mathrm{in}}$ a 5 by 5 random pixel value matrix.

(4) $$G_{\mathrm{in}}=\left[\begin{array}{ccccc} 1 & 2 & 1 & 4 & 3\\ 1 & 2 & 2 & 3 & 4\\ 5 & 7 & 6 & 8 & 9\\ 5 & 7 & 6 & 8 & 8\\ 5 & 6 & 7 & 8 & 9 \end{array}\right]$$

First, take the average of the graphs, and the formula is

(5) $$v=\frac{\sum_{i=1}^{n}{}_{x_{i}}}{n}$$

In the equation, *n* is the total number of pixels in the image, and *x_i_* is the original pixel value. It is concluded that v = 5 (integer). 

Then, average filtering means that the pixel at the center of the graph is the average value of all pixel values, and the formula is

(6) $$G_{\mathrm{mean}}=g(x,y)=\frac{1}{M}\sum_{}f(x,y)$$

In the equation, g(x,y) is the pixel value after filtering, f(x,y) is the pixel value of the original image, and M is the square matrix selected during filtering (generally 3 × 3).

(7) $$f(x,y)=\left[\begin{array}{ccccc} 1 & 2 & 1 & 4 & 3\\ 1 & 2 & 2 & 3 & 4\\ 5 & 7 & 6 & 8 & 9\\ 5 & 7 & 6 & 8 & 8\\ 5 & 6 & 7 & 8 & 9 \end{array}\right]\Rightarrow g(x,y)=\left[\begin{array}{ccccc} 1 & 2 & 1 & 4 & 3\\ 1 & 3 & 4 & 4 & 4\\ 5 & 4 & 5 & 6 & 9\\ 5 & 6 & 7 & 8 & 8\\ 5 & 6 & 7 & 8 & 9 \end{array}\right]$$

Finally, according to Equation (3), subtract f(x,y) from g(x,y) and add v to get $G_{out}$.

(8) $$G_{out}=\left[\begin{array}{ccccc} 5 & 5 & 5 & 5 & 5\\ 5 & 4 & 3 & 4 & 5\\ 5 & 8 & 6 & 7 & 5\\ 5 & 6 & 4 & 5 & 5\\ 5 & 5 & 5 & 5 & 5 \end{array}\right]$$

Step 2: Threshold segmentation. 

It can be seen from Figure 3 that the brightness of the two defects is inconsistent. The defects increased by the cladding layer appear brighter in the image and the stripe defects appear darker in the image. Therefore, a high threshold is set to segment the lighter part of the paving defect, and a low threshold is set to segment the darker part of the laying defect. The threshold segmentation equation is shown as below:

(9) $$G^{\prime }(i,j)=\left\{ \begin{array}{l} 255\;\;\;\;G(i,j)\geq H\;\;or\;\;G\;\;(i,j)\leq L\\ 0\;\;\;\;\;\;\;\;\;L<G(i,j)<H \end{array}\right.$$

In the equation, $G^{\prime }(i,j)$ is the image after threshold division, G(i,j) is the image after uneven illumination processing, H is the high threshold, and L is the low threshold. 

In order to select the appropriate threshold under different lighting environments, this paper sets the high threshold as $u+k_{1}\sigma$ and the low threshold as $u-k_{2}\sigma$, where u is the gray average value of the image, σ is the gray standard deviation, and $k_{}$ is the amplification factor. 

Step 3: Image denoising. 

Because SLM equipment is printed with fine metal powder, the metal powder laid on the powder bed is in a loose state, so the reflection state to the light is different, which will lead to a lot of noise in the powder image. In order to reduce the difficulty of image processing, it is necessary to denoise the powdering image. The image denoising methods used in this paper mainly include morphological filter and connected domain filter. Small noise can be removed by the open operation of morphological filter, and large noise can be removed by connected domain filter. 

For the defects increased in the cladding layer, the open operation of image morphology processing is carried out for denoising, that is necessary to use 3 × 3 structural elements to perform the opening operation, so that the pixel area containing 3 × 3 can be retained, and finally the area smaller than 30 pixels is deleted through the connected domain filter to extract this defect. 

For the stripe defects, it is necessary to perform open operations on the image using 3 × 3 structural elements and 1 × 5 structural elements, and then merge the two open operation images, and then use 2 × 20 structural elements to perform expansion operations. Finally, the region with area less than 1500 pixels or width less than 150 pixels should be deleted through connected domain filter, so that the defect can be extracted. 

**Figure 5:**
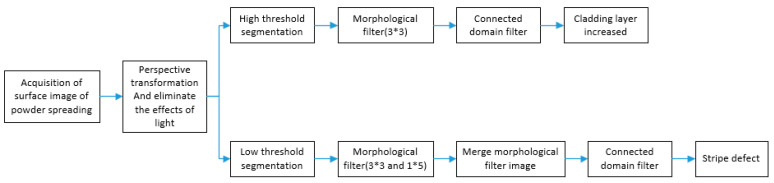
The flow chart of powder defect extraction.

**Figure 6:**
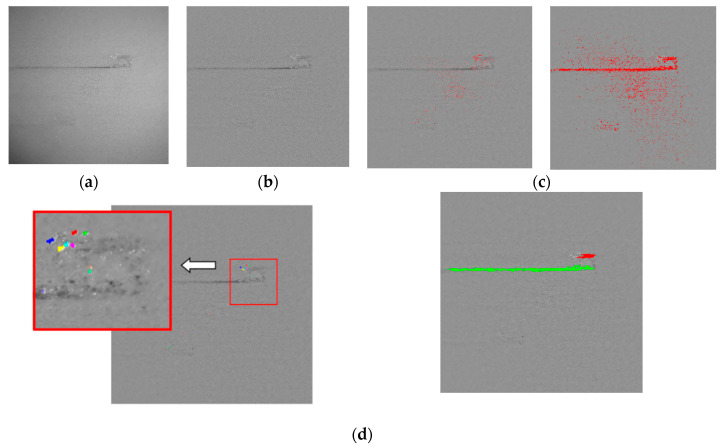

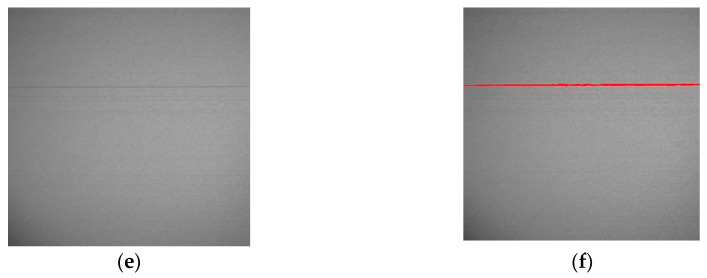
The defect extraction effect: (**a**) Original image. (**b**) Step 1. (**c**) Step 2: The image on the left is high threshold, and the image on the right is low threshold. (**d**) Step 3: The image on the left is cladding defect, and the image on the right is stripe defect. (**e**) Original image. (**f**) The extraction results.

The flow chart of powder defect extraction is shown in Figure 5. Taking Figure 3 as an example, the defect extraction effect is shown in Figure 6. 

## 4. Stripe Defect Classification

As the stripe defects include impurity defects and scraper damage defects, these two types of defects need to be classified. The Halcon machine vision software package provides two classifiers with fast detection speed: the multilayer perceptron and support vector machine. The two classifiers will be analyzed below. 

### 4.1. The Multilayer Perceptron (MLP)

The multilayer perceptron (MLP) classifier is mainly based on an artificial neural network and is composed of multiple single-layer perceptron. Generally, the backpropagation algorithm is used to train the neural network. The classifier has excellent nonlinear mapping ability, adaptive ability, generalization ability, and fault tolerance ability, but the convergence speed of the whole algorithm is slow, leading to a long training time. 

In this paper, the minimum pixel value, pixel standard deviation, defect height, and defect roundness value of stripe defects are used as feature vectors. Therefore, the number of neurons in the input layer of the multilayer perceptron network structure is set to four, and the number of neurons in the output layer is set to two. The maximum-minimum method is used for data normalization, and the excitation function is selected as softmax. At present, there is no unified theoretical basis for determining the optimal solution for the number of hidden layer neurons in a multilayer perceptron. In this paper, the number of neurons in the hidden layer is determined as two by empirical equation five.

(10) $$H=\log_{2}n$$

In the equation, *H* is the number of hidden layer neurons and *n* is the number of input layer neurons. 

In order to verify the classification effect of the multilayer perceptron, 40 images of impurity defects and 40 images of scraper damage defects were used as training samples to train MLP. After the training, stripe defects were classified. The test samples contained 20 impurity defect samples and 20 scraped damage defect samples. At the same time, in order to verify the effect of defect-free and cladding layer increased defects, another 20 qualified samples and 20 cladding layer increased defect samples were added. The confusion matrix of MLP classification results are shown in Table 1. 

### 4.2. Support Vector Machine (SVM)

Support vector machine (SVM) classifier using structural risk minimization principle can effectively solve the problem of small batch sample of learning, The idea is to find a partition hyperplane that can distinguish different samples in the training sample space. For nonlinear data, it can be mapped into a high-dimensional space through a kernel function, and finally find the support vector and seek the maximum interval hyperplane. The final decision function of the classifier is only determined by the key support vectors. To a certain extent, it can effectively avoid the dimensional disaster and the classification speed is fast, but the effect of SVM classifier for large-scale training samples will be greatly reduced. 

The SVM is trained with the same feature vector and training samples, and the same test samples are used for classification. The confusion matrix of SVM classification results are shown in Table 2. 

## 5. Comparison with Other Methods

### 5.1. Photodiode

Photodiode is a kind of photoelectric sensor that can convert an optical signal into an electrical signal [21]. It can output the corresponding analog electrical signal according to the illumination of the received light or realize the switch between different states in the digital circuit. The precision of photodiode data is related to the distance between the pool and the diode, incident angle, etc. Moreover, the higher the laser power is, the larger the fluctuation range of photodiode signal value is, and the worse the stability of the pool. Compared with photodiode, our method does not need to strictly adjust the distance angle, only needs to display the molding process in the camera, and is not affected by the laser power [22]. 

### 5.2. X-ray

X-ray can intuitively reflect the three-dimensional morphology and position of internal defects. Hu et al. [23] characterized the porosity and poor fusion defects by X-ray computer, predicted the fatigue life by combining with the fatigue crack propagation model, and effectively judged the fatigue threat level caused by different positions of defects. Although X-ray can directly monitor the morphology and location of internal defects in real-time, X-ray monitoring has a high cost, which is harmful to the human body and requires additional protection [24]. In particular, the cost of high-speed and high-resolution X-ray in situ observation is higher, which is generally used for checking and verifying other monitoring methods. In contrast, the use of industrial cameras has a lower cost. 

### 5.3. Thermal Signal

Heat transfer is a driving force of the SLM, including the formation and dynamic behavior of molten pool, the liquid metal cooling and solidification, solidification layer of thermal cycle, microstructure, residual stress, and deformation of component has a direct impact, such as uniform temperature distribution form good quality components, unreasonable temperature distribution will affect the structural integrity and quality. However, at present, the difficulty of temperature detection is that the emissivity of the material is difficult to obtain, and in the SLM process, the form of the material includes the powder state, liquid state, solid state, and gas state, and the emissivity is also changing due to the difference of temperature at different positions. Therefore, it is very difficult to obtain the emissivity in SLM process [25]. 

### 5.4. Vibration Signal

The vibration signal in the SLM process can also reflect the processing state and component quality, such as penetration depth, crack, powder laying quality, etc. However, the research on SLM process monitoring based on vibration signals is few at present, and the selection, arrangement, signal collection, and processing of sensors need to be further studied [25]. 

## 6. Discussion

From Figure 3 and Figure 4, it is not difficult to calculate that the recognition rates of MLP and SVM are 98.33% and 97.5%, respectively. Both have high recognition rate, but MLP recognition rate is better than SVM. 

**Figure 7:**
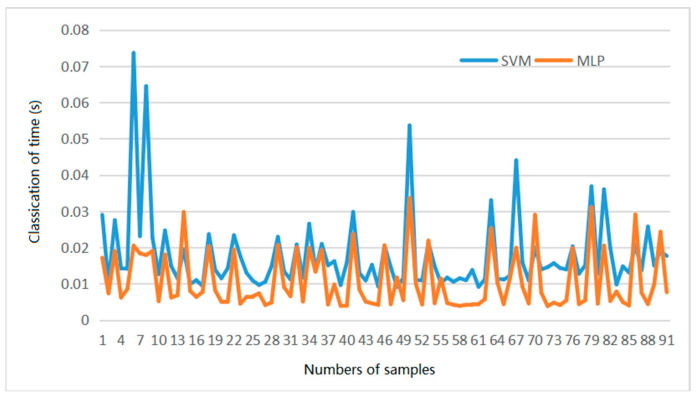
Classification time of MLP and SVM.

In addition, the classification time will increase the forming time of SLM equipment and reduce the forming efficiency. Therefore, the classification time of the two classifiers should be discussed; Figure 7 is the classification time comparison diagram of MLP and SVM. As you can see from Figure 7, the MLP classification time is shorter. 

In summary, MLP has a better classification performance than SVM, so MLP is chosen to classify stripe defects. 

## 7. Conclusions

This paper presents a method for detecting powder spreading defects and monitoring the powder spreading surface as well as providing corresponding hardware construction measures and image processing methods. Using this method, we accurately established the relationship between image features and these three defects. This method first eliminates linear distortion through perspective transformation and uses Equation (3) to eliminate lighting impact, followed by selecting a high threshold and a low threshold to extract the defects increased by the cladding layer and the stripe defects with image noise, and then filtering the image noise through the morphological filter algorithm and the connected domain filter algorithm to obtain the accurate area of the powder spreading defects. Finally, a multilayer perceptron and a support vector machine were established to classify the stripe defects. The two classifiers were compared, and the multilayer perceptron with better performance was selected as the final classifier. The detection method proposed in this paper has high recognition rate, short detection time, and good stability. Subsequently, the corresponding feedback operations can be designed according to their corresponding defect types. The study on choosing type defect resolution and recognition has achieved good effect and can be immediately applied to the industrial technology of ascension, but due to the use of industrial camera, the image resolution limit is 0.5 mm, so for defects of smaller size, establishing the rate of detection and the relationship between the image characteristics and other defects are key problems to solve in the future. Through this research, we can apply machine vision to the laser cladding of diamond, monitor the formation of the graphite interface of the diamond–substrate interface, adjust the laser spot through feedback, prevent the diamond from graphitization, and better fix the diamond; machine vision can also be applied Based on laser metal cladding technology, monitoring the thickness of the coating, feedback and adjusting the parameters to obtain a thinner effective coating to protect the base metal, and so on. It can be seen that machine vision technology can be combined with various engineering and processing methods and has a wide range of applications in the industrial field.

## Figures and Tables

**Table 1:** The confusion matrix of the MLP classification results.

	Predicted Class			
Actual Class	Qualified	Cladding Layer Increased Defects	Impurity Defects	Scraper Damage Defects
Qualified	19	1	0	0
cladding layer increased defects	0	20	0	0
Impurity defects	0	0	39	1
Scraper damage defects	0	0	0	40

**Table 2:** The confusion matrix of the SVM classification results.

	Predicted Class			
Actual Class	Qualified	Cladding Layer Increased Defects	Impurity Defects	Scraper Damage Defects
Qualified	19	1	0	0
cladding layer increased defects	0	20	0	0
Impurity defects	0	0	38	2
Scraper damage defects	0	0	0	40

## Data Availability

The raw data supporting the conclusions of this article will be made available by the authors, without undue reservation.
